# Relocation of the attTn7 Transgene Insertion Site in Bacmid DNA Enhances Baculovirus Genome Stability and Recombinant Protein Expression in Insect Cells

**DOI:** 10.3390/v12121448

**Published:** 2020-12-16

**Authors:** Gorben P. Pijlman, Carissa Grose, Tessy A. H. Hick, Herman E. Breukink, Robin van den Braak, Sandra R. Abbo, Corinne Geertsema, Monique M. van Oers, Dirk E. Martens, Dominic Esposito

**Affiliations:** 1 Laboratory of Virology, Wageningen University, Droevendaalsesteeg 1, 6708PB Wageningen, The Netherlands; tessy.hick@wur.nl (T.A.H.H.); herman.breukink@agilent.com (H.E.B.); robinvandenbraak@hotmail.com (R.v.d.B.); sandra.abbo@wur.nl (S.R.A.); corinne.geertsema@wur.nl (C.G.); monique.vanoers@wur.nl (M.M.v.O.); 2 Protein Expression Laboratory, Cancer Research Technology Program, Frederick National Laboratory for Cancer Research, Leidos Biomedical Research, Inc. PO Box B, Frederick, MD 21702, USA; carissa.grose@nih.gov (C.G.); dom.esposito@nih.gov (D.E.); 3 Bioprocess Engineering, Wageningen University, Droevendaalsesteeg 1, 6708PB Wageningen, The Netherlands; dirk.martens@wur.nl

**Keywords:** baculovirus, insect cells, bacmid, Tn7, genome stability, protein expression, chikungunya virus, VLPs, bioreactor

## Abstract

Baculovirus expression vectors are successfully used for the commercial production of complex (glyco)proteins in eukaryotic cells. The genome engineering of single-copy baculovirus infectious clones (bacmids) in *E. coli* has been valuable in the study of baculovirus biology, but bacmids are not yet widely applied as expression vectors. An important limitation of first-generation bacmids for large-scale protein production is the rapid loss of gene of interest (GOI) expression. The instability is caused by the mini-F replicon in the bacmid backbone, which is non-essential for baculovirus replication in insect cells, and carries the adjacent GOI in between attTn7 transposition sites. In this paper, we test the hypothesis that relocation of the attTn7 transgene insertion site away from the mini-F replicon prevents deletion of the GOI, thereby resulting in higher and prolonged recombinant protein expression levels. We applied lambda red genome engineering combined with SacB counterselection to generate a series of bacmids with relocated attTn7 sites and tested their performance by comparing the relative expression levels of different GOIs. We conclude that GOI expression from the *odv-e56* (*pif-5*) locus results in higher overall expression levels and is more stable over serial passages compared to the original bacmid. Finally, we evaluated this improved next-generation bacmid during a bioreactor scale-up of Sf9 insect cells in suspension to produce enveloped chikungunya virus-like particles as a model vaccine.

## 1. Introduction

The baculovirus expression vector system (BEVS) is one of the most versatile platform technologies developed in the past 30 years to produce complex (glyco)proteins in eukaryotic cells, because it frequently results in high yields of purified proteins and allows for similar post-translational modifications as mammalian expression systems [1]. An increasing number of commercial BEVS products are becoming available, and these include human vaccines against influenza A virus and human papilloma virus, gene therapy products, and veterinary vaccines [2,3]. More BEVS bio-pharmaceuticals are currently in (pre)clinical trials and these include prototype COVID-19 vaccines that are aimed at protection against SARS-CoV-2 infection [4]. Traditional baculovirus expression vectors are generated by homologous recombination in insect cells between wildtype baculovirus DNA and a so-called transfer vector (plasmid DNA), which carries the gene of interest (GOI) under a suitable baculovirus promoter, typically the very strong polyhedrin or p10 promoter [5,6]. There have been sophisticated improvements throughout the years to streamline the generation and isolation of recombinant baculoviruses, and numerous easy-to-use commercial baculovirus kits are available [7].

**Figure 1:**
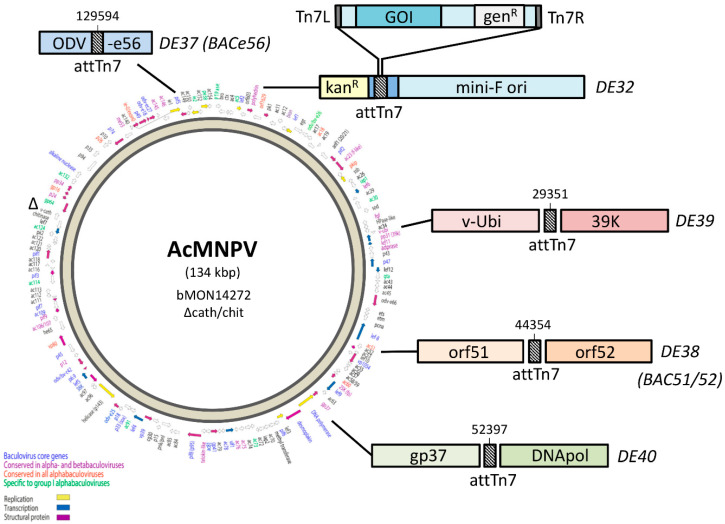
Bacmid strains with relocated attTn7 sites. Schematic diagram of new attTn7 insertion sites in the modified bacmid strains. The crosshatched boxes represent the attTn7 sites introduced into different loci in the parental DE32 bacmid. Insertion site location numbering is based on the wildtype *Autographa californica* multiple nucleopolyhedrovirus (AcMNPV) E2 genome sequence.

One of the major breakthroughs in baculovirus technology was the generation of an *Autographa californica* multiple nucleopolyhedrovirus (AcMNPV) infectious clone that could be maintained as a ~140 kb large, single-copy bacterial artificial chromosome (BAC) in *E. coli* [8]. This so-called “bacmid” carries a mini-F replicon for single-copy replication in *E. coli*, a kanamycin resistance gene (kan^R^) for selection, and a LacZ-attTn7 transposon integration site (inserted between kan^R^ and mini-F) for insertion of the GOI by Tn7 transposition (Figure 1). A commercial kit based on these bacmids was successfully launched by Invitrogen Life Technologies (now ThermoFisher) as the “Bac-to-Bac baculovirus expression system”, which uses so-called “pFastBac” transfer vectors in which the GOI is cloned in between attL and attR sites, and downstream of the strong polyhedrin (polh) or p10 promoter. After Tn7 transposition, the recombinant bacmids are selected for antibiotic resistance (kan^R^ and gen^R^) and, upon transfection of insect cells, this yields, at least in theory, a genetically homogeneous recombinant baculovirus stock that does not require further plaque purification. 

While bacmids are efficient for rapidly creating recombinant baculoviruses, the introduction of genes is limited to a single insertion site. MultiBac (Geneva Biotech) has improved the system to allow for the introduction of several genes, with the multigene construct being shuttled into the same attTn7 site [9]. The original AcMNPV bacmid (bMON14272, [8]) can be easily manipulated in *E. coli* and this has been the driving force for initiating numerous knock out studies to study baculovirus gene functions. Bacmids have been constructed for baculoviruses other than AcMNPV [10,11,12] and this has tremendously accelerated functional studies on previously uncharacterized baculovirus genes, including baculovirus core genes or those involved in oral infectivity [13,14,15,16]. 

Despite the major contribution of bacmids in the understanding of baculovirus biology, most commercial baculovirus vectors are still based on the traditional method of homologous recombination in insect cells between a transfer plasmid carrying the GOI and a (linearized) AcMNPV genome, followed by several rounds of plaque purification and master seed production.

For large-scale protein expression, bacmid-based expression vectors have not been applied, despite improvements that have increased the efficiency of GOI transposition and recombinant bacmid selection [17]. This may change soon, however, since Novavax appears to produce its COVID-19 vaccine NVX-CoV2373 using a pFastBac vector containing a SARS-CoV-2 spike gene [18]. An important reason for not using bacmids for large-scale production is the relative rapid loss of GOI expression upon serial passage [19] resulting from the genomic instability of the mini-F replicon in the bacmid backbone [20]. The exact reason for the rapid deletion of the mini-F and the adjacent GOI is unknown, but its bacterial origin and large size (>8 kbp) likely account for a negative selection pressure on recombinant baculovirus genomes during replication in insect cells. 

We hypothesize that relocation of the attTn7 away from the mini-F replicon would prevent or at least delay GOI deletion, thereby resulting in higher recombinant protein expression levels upon serial passage. To test this hypothesis, we generate a series of bacmids with attTn7 insertions at different locations on the AcMNPV genome that had previously been shown to tolerate insertions [21]. We analyze the performance of the different bacmids by comparing the relative expression levels of different GOIs. The best performing bacmid is subsequently tested for the stability of recombinant protein expression upon serial undiluted passages in insect cells. Finally, we demonstrate the viability of this novel bacmid for industrial vaccine scale-up by producing chikungunya virus-like particles (CHIKV VLPs) in Sf9 suspension cells in shaker flasks and a bioreactor [22,23,24,25].

## 2. Materials and Methods

### 2.1. Construction of ΔattTn7 Bacmid

Before moving the attTn7 binding region to alternate locations, the original attTn7 site had to be removed. To knock out the attTn7 binding region, a linear cassette was generated by a Gibson Isothermal Assembly, which included ~300 bp homology arms in both the pLac and LacZ regions along with the counter-selectable cassette Cat-SacB (chloramphenicol acetyltransferase-sucrose sensitivity), which was amplified from pELO4 (gift from Don Court, NCI); both homology arms included 40 bp of sequence homology at the appropriate ends of the Cat-SacB cassette. Twenty-five femtomoles of each amplicon were included in a 20 μL reaction with 2× Gibson Assembly Master Mix (New England Biolabs, Ipswich, MA, USA) and incubated at 50 °C for 30 min. To amplify the assembled cassette, 1 μL of the assembly reaction was used as a template in a 100 μL PCR reaction, including 0.4 μM of each primer and 2× Phusion HF Mastermix (60 °C annealing temperature, 4 min extension, 25 cycles). The linear cassette was purified using a QiaQuick PCR Purification Kit (Qiagen, Hilden, Germany).

The original strain for this work was λRed *E. coli* SW106 (gift from Don Court, NCI) harboring an AcMNPV bacmid, from which the chitinase and cathepsin genes had previously been deleted (bMON14272Δvcath-chiA or DE32). The strain was grown in LB with 50 μg/mL kanamycin at 30 °C with shaking to OD_600_ of 0.4. The culture was agitated in a 42 °C water bath for 15 min to induce the temperature-sensitive lambda prophage. The cells were then chilled and made electrocompetent. One hundred nanograms of the knock-out cassette were added to 50 μL of the cells, which were subsequently electroporated. The transformation was incubated with shaking for two hours at 30 °C in 1 mL LB before plating 100 μL on LB agar with 50 μg/mL kanamycin and 20 μg/mL chloramphenicol at 30 °C. Four colonies were selected on the second morning and diluted in 50 μL water, with 1 μL being used as a template for colony PCR with primers that bound just outside the insertion. 

Once this intermediate bacmid was confirmed, the Cat-SacB selection cassette was seamlessly removed. Similar ~300 bp arms, homologous to the end of pLac and the beginning of LacZ, were generated by PCR, where the LacZ arm product included 40 bp homology to the end of pLac. The two amplicons were assembled by overlap PCR (0.2 μL of each amplicon, 2× HF mastermix, 0.4 μM primers, 100 μL total volume) (60 °C annealing, 30 s extension, 25 cycles) to produce the marker knock-out cassette and were purified using a QiaQuick PCR Purification Kit. Lambda Red was induced in the SW106 bMON14272Δvcath-chiAΔ*attTn7*:Cat-SacB strain and electrocompetent cells were made as described above. One hundred nanograms of the marker removal cassette was added to 50 μL of electrocompetent cells and transformed by electroporation. The cells were allowed to recover by shaking at 30 °C for 4 h in 10 mL LB. Cells from 1 mL of the transformation were pelleted by centrifugation for 30 s at 13,000× *g* and resuspended in 1 volume 1× M9 salts twice to wash the cells. After the second wash, the cells were resuspended in 100 µl M9 salts and plated on LB agar with no salt, 6% sucrose, and 50 µg/mL kanamycin at 37 °C, to select cells that no longer contained the Cat-SacB selection marker. Bacmid DNA was isolated from four colonies by alkaline lysis and the recombined region was amplified by PCR to confirm the deletion of the Cat-SacB marker. The resulting bacmid with the correctly sized amplicon was verified by sequencing the entire cloned region. 

The confirmed bMON14272Δvcath-chiAΔ*attTn7* was used to transform *E. coli* DE25 cells, a DH10B-derived cell line compatible with the Bac-to-Bac system for recombinant baculovirus production. The strain contains an optimized helper plasmid that encodes Tn7 transposition functions to generate recombinant baculovirus and confers resistance to tetracycline [17]. The transformation was plated on LB with 50 µg/mL kanamycin and 12.5 µg/mL tetracycline at 37 °C. A single colony was selected from the plate to generate competent cells for the new deletion strain.

### 2.2. Construction of Bacmids with Relocated attTn7

Bacmids with relocated attTn7 sites Strains DE37, DE38, DE39, and DE40 were generated in a similar fashion. The knock-in cassettes for odv-e56 (pif-5) and the intergenic regions of orf51/52, v-ubiquitin (v-ubi)/39 k, and gp37/DNApol included 4 amplicons, which contained a ~300 bp region of homology to the immediate left of the desired insertion point with a 40 bp homology to attTn7 (Forward: 5′- TGTGGAATTGTGAGCGGATA; Reverse: 5′-TCCTGTGACGGAAGATCACTTCGCAGAATAAAT AAATCCTGGTGCTGCAAGGCGATTAAGT), Cat-SacB cassette, and ~300b p region of homology to the right of the insertion including 40 bp homology to the end of SacB (Table 1). 

The amplicons were assembled by Isothermal Assembly with 25 femtomoles of each piece in a 20 μL reaction with 2× Gibson Assembly Master Mix (New England Biolabs) and incubated for 30 min at 50 °C. The cassette was then amplified with 1 μL of the assembly reaction as a template in a 100 uL PCR reaction, including 0.4 μM of each primer and 2× Phusion HF Mastermix (60 °C annealing temperature, 4 min extension, 25 cycles). The linear cassette was purified using a QiaQuick PCR Purification Kit (Qiagen).

The previously generated strain, SW106 bMON14272Δvcath-chiA; ΔattTn7, was induced as described previously and electrocompetent cells were made. One hundred nanograms of the attTn7 knock-in cassette was added to 50 μL of electrocompetent cells and electroporated at 1.8 kV. The cells were recovered in 1 mL LB at 30 °C for 2 h and 100 μL was then plated on LB with 50 ug/mL kanamycin and 20 ug/mL chloramphenicol. Four colonies were selected on the second morning and diluted in 50 uL water, with 1ul being used as template for colony PCR, with primers landing just outside the insertion. The remaining diluted cells were then used to seed cultures to generate glycerol stocks for the selected positive construct. The Cat-SacB marker was removed from these intermediate strains in a manner similar to that described above. The loss of the marker was confirmed by PCR amplification of the insertion region and sequencing of the entire region for each new bacmid. 

The confirmed bacmids were transformed into *E. coli* DE25 cells as described above. A single colony was selected from each plate to generate competent cells for DE37, DE38, DE39, and DE40. The entire bacmid for each strain was analyzed by PacBio sequencing to be certain that there was no unintended rearrangement during recombination.

### 2.3. Generation of Recombinant Baculoviruses

To test the expression levels at each location, four GOIs were tested: mouse A15 extracellular domain (NP_062608.2), human NDUFA13 (NP_057049.5), human FLCN (NP_659434.2), and enhanced green fluorescent protein (eGFP). Expression clones were generated for each by gateway recombination of previously validated entry clones into baculovirus expression backbones (pDest-636), including the polh promoter and N-terminal His6-MBP (maltose-binding protein) tag. To facilitate shuttling into the bacmid, the expression backbone contained Tn7 left and right arms as well as the gentamycin resistance marker. One microliter of the verified expression clone was used to transform 50 μL of chemically competent cells of each of the new strains (DE37-DE40) as well as the DE32 (Δvcath-chiA) control. One ml of LB was added to the transformants and cultures were allowed to grow at 37 °C for 4 h with shaking. Ten microliters of the outgrowth were diluted into 190 μL LB and the resulting 200 μL was plated onto LB agar plates with gentamycin (7 ug/mL), kanamycin (50 ug/mL), tetracycline (10 ug/mL), IPTG (40 ug/mL), and Bluo-gal (100 ug/mL), and grown overnight at 37 °C. White colonies were selected from each plate and grown overnight in 3 mL LB with gentamycin and kanamycin at 37 °C with shaking. Two milliliter aliquots were pelleted and bacmid DNA was prepared by alkaline lysis. Junction PCR was performed to verify the correct inserts using the lysis as template. Fifty milliliters of Sf9s at a density of 1.5 × 10^6^ cells/mL was transfected with 25 μL of bacmid complexed with 125 μL of Insect GeneJuice (Sigma-Aldrich, Saint Louis, MO, USA). The complexes were allowed to form for 20 min and were then added to the culture. The cultures were incubated for 5 days and then harvested by centrifugation at 2000 RPM for 10 min and the supernatants were then collected in 50 mL conical tubes.

### 2.4. Recombinant Protein Production and Quantitation

For small-scale protein expression, 50 mL of Tni-FNL cells were seeded at 1.5 × 10^6^ cells/mL and were infected with the generated viruses at a multiplicity of infection (MOI) of 3. The infected cultures were allowed to grow at 27 °C for 72 h. The cells were then harvested by centrifugation at 2500 rpm for 20 min in 50 mL conical tubes. The pellets were each resuspended in 5 mL buffer (20 mM Hepes pH 7.3, 300 mM NaCl, 1 mM TCEP) by gently vortexing, and then were lysed using an LV-1 microfluidizer (Microfluidics, Inc., Westwood, MA, USA) set at a pressure of 7000 psi for 2 passes per lysate. Lysates were then clarified using ultracentrifugation at 33,100 rpm for 30 min, and the supernatant was collected in a 15 mL conical tube. Clarified cell extracts of A15, NDUFA13, and FLCN were purified using immobilized metal affinity chromatography on Phynexus tips with elution in 500 mM imidazole. Final proteins were analyzed by SDS–PAGE chromatography to ensure purity, and quantitated in a Nanodrop spectrophotometer at 280 nm. Cell extracts expressing eGFP were quantified by measuring GFP fluorescence (excitation = 485 nm, emission = 510 nm) on a BMG Omega plate reader. Data presented are averages of three replications of the purification process with the standard deviation shown via error bars.

### 2.5. Serial Undiluted Baculovirus Passage on Sf21 Cells

Bacmid-derived viruses expressing CHIKV VLPs were passaged ten times in duplicate in Sf21 cells, as described before [19,25]. Sf21 cells were cultured in supplemented Grace’s insect medium (Gibco, Life Technologies, Carlsbad, CA, USA) with 10% fetal bovine serum (FBS, Invitrogen, Carlsbad, CA, USA) and 50 μg/mL gentamycin (Gibco, Life Technologies) in a T25 cell culture flask (Greiner, Alphen aan den Rijn, the Netherlands). For serial passage, 1 mL of each virus suspension was added to a T25 flask that contained healthy Sf21 cells with a confluency of 50–60%. After three hours, the virus suspension was replaced with 4 mL fresh medium. The cells were incubated for 3 days at 27 °C. Cells were detached and the cell suspension was centrifuged for 5 min at 4000 rpm. The cell pellet was washed once with 500 µL PBS and resuspended in PBS with protease inhibitor (Roche, Basel, Switserland). For the next virus passage, 1 mL of the supernatant was added to new healthy Sf-21 cells. The remaining supernatant was stored at 4 °C. This procedure was repeated ten times in duplicate for each virus.

### 2.6. SDS–PAGE and Western Blot Immunodetection

Protein samples containing equal numbers of cells were analyzed in 12.5% SDS–PAGE gels (Mini-PROTEAN^®^ Tetra System, Bio-Rad Laboratories, Hercules, CA, USA). Proteins were transferred to an Immobilon-P membrane (Millipore) using a Tris–Glycine buffer (25 mM Tris, 192 mM glycine, 10% (*v*/*v*) methanol, pH 8.3). Membranes were blocked in 3% low-fat milk powder (Campina, The Netherlands) in PBS 0.1% (*v*/*v*) Tween-20 (PBS-T) (Merck). Blots were washed with 2.5 mL PBS-T for 5 min at RT and the primary polyclonal antibody rabbit-anti-CHIKV-E2 or -E1 immunoglobulin was added (dilution 1:10,000). For detection of the AcMNPV VP39 major capsid protein, a polyclonal mouse anti-VP39 immunoglobulin was used (dilution 1:1000). Secondary antibodies were polyclonal goat anti-rabbit immunoglobulin (AP-conjugated) or polyclonal goat anti-mouse immunoglobulin (AP-conjugated) (both Sigma-Aldrich). After incubation and washing, the blot was stained with NBT/BCIP (Roche Diagnostics GmbH, Basel, Switserland).

### 2.7. Shake Flask and Bioreactor Experiments

In the shake flask experiments, Sf9 suspension cells were cultured in Sf-900 II SFM medium (Gibco, Life Technologies, Carlsbad, CA, USA) in 125 mL flasks (Nalgene, Rochester, NY, USA) with a working volume of 25 mL. The culture conditions of the cells were identical to the culture conditions of the maintenance culture (27 °C, 100 rpm, inoculation at 0.5 × 10^6^ cells/mL from an exponential phase cell culture at ~4 × 10^6^ cells/mL). When the desired cell concentration was reached in the shake flasks, infection with the recombinant baculovirus was performed at a defined MOI. Samples were obtained at specific time points to analyze the infection process. A 1-litre DASGIP bioreactor (Eppendorf, Hamburg, Germany) was inoculated at 0.5 × 10^6^ cells/mL by the Sf9 culture stock in the exponential growth phase. The bioreactors had a working volume of 500 mL and cells were maintained in Sf-900 II SFM medium (Gibco, Life Technologies, Carlsbad, CA, USA) at 27 °C and an agitation speed of 150 rpm. The DO (DO sensor, Broadley James, Irvine, CA, USA) was controlled at 30% air saturation by headspace aeration. The pH (pH sensor, Mettler Toledo, Tiel, the Netherlands) was retained at a value of 6.3 by automatic base and CO_2_ addition. Once the cells reached the desired CCI, infection with recombinant baculoviruses was performed at a defined MOI. Samples were obtained at several time points during the infection process.

## 3. Results

### 3.1. Construction of AcMNPV Bacmids with a Relocated attTn7 Integration Site

**Figure 2:**
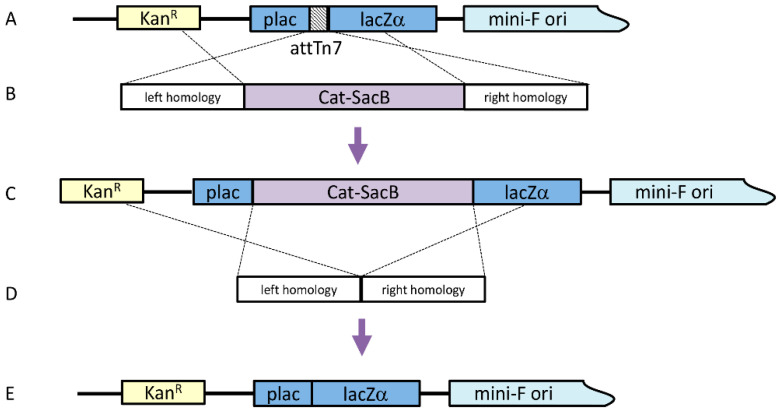
Construction of ΔattTn7 bacmid. (**A**) Parent strain; (**B**) linear attTn7 removal cassette; (**C**) intermediate Cat-SacB strain; (**D**) linear removal marker; (**E**) final strain.

Four different bacmids were constructed, each with the attTn7 integration site at different locations in the AcMNPV genome (Figure 1). A modified bacmid lacking the chitinase/cathepsin loci was used as a parental bacmid (bMON14272Δvcath-chiA or DE32). First, the attTn7 site was removed from its original location (polh locus) by a two-step homologous recombination in *E. coli* (Figure 2). 

Briefly, a Cat-SacB positive/negative selection cassette was generated by PCR and electroporated into *E. coli* harboring DE32 (Figure 2A,B). Chloramphenicol-resistant colonies were selected (Figure 2C) and another PCR product, consisting of attTn7 flanking regions, was then introduced by electroporation (Figure 2D). Bacmids were counterselected against SacB and the resulting bacmids containing a scarless deletion of attTn7 were selected (Figure 2E). 

**Figure 3:**
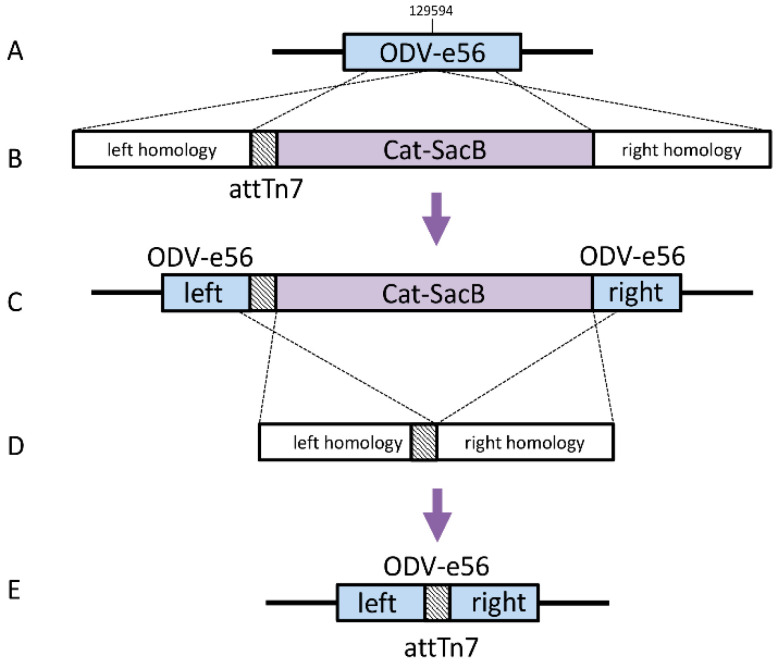
Construction of bacmids with relocated attTn7. (**A**) Parent strain; (**B**) linear attTn7 removal cassette; (**C**) intermediate Cat-SacB strain; (**D**) linear removal marker; (**E**) final strain.

Next, the attTn7 site was inserted by homologous recombination at four different loci in the AcMNPV genome: in the odv-e56 ORF (DE37 or BACe56), in between orf51 and orf52 (DE38 or BAC51/52), in between v-Ubi and 39k (DE39) and in between DNApol and gp37 (DE40) (Figure 3).

### 3.2. Heterologous Protein Expression Levels with Modified Bacmids

**Figure 4:**
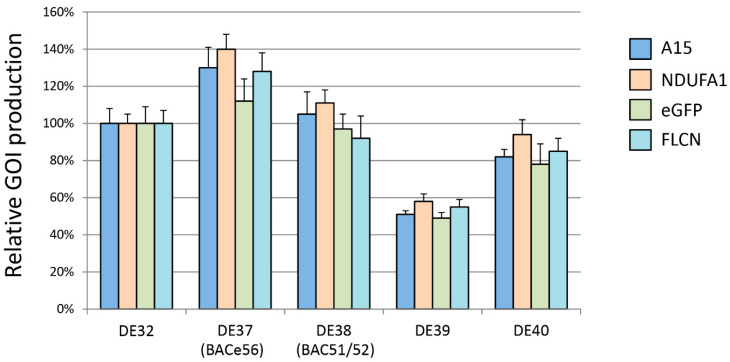
Protein expression in Tni-FNL cells using modified bacmid strains. Protein expression in the modified bacmid strains was measured by green fluorescent protein (GFP) fluorescence (eGFP) or protein quantitation of small-scale IMAC-purified proteins (A15, NDUFA1, FLCN). In all cases, the protein levels were normalized to the level of protein produced in the DE32 control strain. Data represent triplicate measurements and standard deviation is noted with error bars.

After the attTn7 site had been relocated, four different heterologous genes known to be challenging to produce in *E. coli* were inserted into the four new bacmids. Recombinant baculoviruses were generated and cells were infected with an MOI of 3 tissue culture infective doses of 50% (TCID_50_)/cell. After 72 h, cells were harvested and protein was purified from a cell extract. Protein levels relative to those of the parental bacmid were assessed (Figure 4). Two of the new bacmids (DE39 and DE40) with the attTn7 inserted in between gp37 and DNApol, and in between v-Ubi and 39 k, displayed an overall lower expression level for all GOIs than the parental bacmid (DE32). The new bacmid with attTn7 inserted in between ORF51 and ORF52 (DE38 or BAC51/52) had a similar expression (90–110%) as the parental bacmid, whereas the new bacmid with attTn7 inserted in the odv-e56 ORF (DE37 or BACe56) displayed enhanced expression levels (110–140%) for all tested GOIs. 

### 3.3. Performance of Novel Bacmids Expressing Chikungunya VLPs upon Serial Undiluted Passage

**Figure 5:**
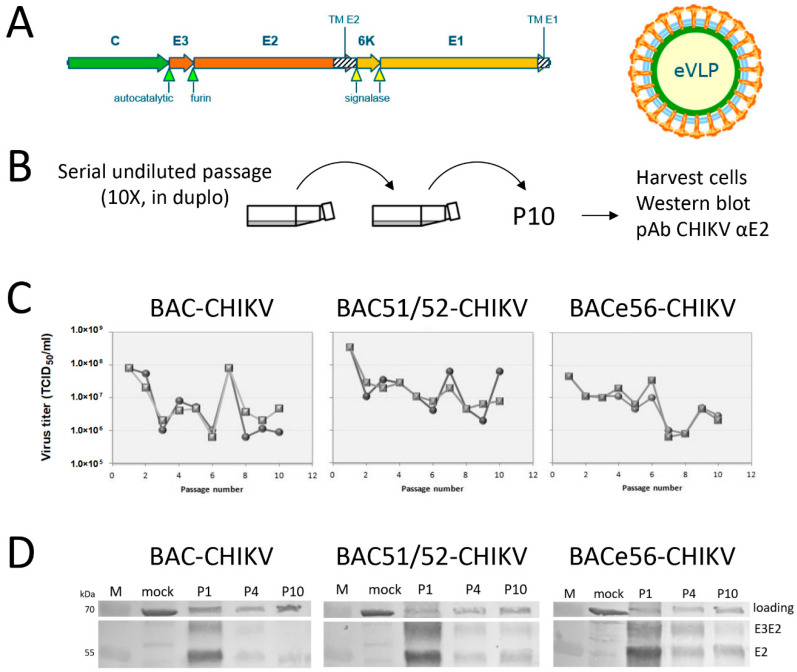
Effect of serial undiluted baculovirus passage on chikungunya virus-like particle (VLP) production. (**A**) Schematic representation of the chikungunya virus (CHIKV) structural genes for insect cell expression of enveloped (e)VLPs using recombinant baculoviruses. Shaded areas represent transmembrane domains. Arrows indicate protease cleavage sites. (**B**) Experimental set-up for serial undiluted baculovirus passage in Sf21 insect cells. (**C**) Viral titers of serial undiluted passaging experiment. Each recombinant baculovirus was passaged in duplicate for 10 passages. Viral titers were determined by end point dilution assay and are expressed as tissue culture infective dose 50% per ml (TCID_50_/_mL_). (**D**) Chikungunya VLP expression upon serial passage. Western blots detected with anti-E2 polyclonal antiserum show CHIKV glycoprotein E2 and the precursor E3E2. M: protein marker; loading: host cell protein; mock: healthy cells.

The two bacmids (BAC51/52 and BACe56) with expression equal to or higher than their parental bacmid were investigated for the stability of heterologous protein expression during serial undiluted passages. As GOI, we chose to express an enveloped virus-like particle (VLP) of chikungunya virus (CHIKV), since this is a glycoprotein-containing complex, capable of self-assembly and inducing a protective immune response in animal trials [22,23,25]. The CHIKV structural genes (capsid, envelope proteins E3, E2, 6K and E1) were expressed from the polh promoter in order to produce CHIKV-enveloped VLPs (Figure 5A) [25]. The recombinant baculoviruses (BAC-CHIKV, BAC51/52-CHIKV and BACe56-CHIKV) were generated by bacmid transfection of Sf21 cells. Serial undiluted passages in Sf21 cells were conducted ten times and in duplicate for each construct (Figure 5B). CHIKV VLP expression was measured by Western bot analysis using an antibody specific to the structural glycoprotein E2, which also recognizes the uncleaved precursor E3E2 [22,26]. The results show that viral titers fluctuated between 10^6^ and 10^8^ TCID_50_/_mL_ during serial undiluted passages (Figure 5C). This is similar to what was seen in other studies [19,27]. Cell lysates of selected passages (P1, P4 and P10) were checked for GOI expression levels (Figure 5D).

Equal amounts of protein were loaded for all samples, and the CHIKV structural proteins E3E2 and E2 were detected by Western blot analysis. For BAC-CHIKV, the expression level of CHIKV (E3)E2 was high at P1, but dramatically decreased at P4 and P10. BAC51/52-CHIKV also had high expression levels at P1, but this was reduced at P4 and P10, although not as low as for BAC-CHIKV. BACe56-CHIKV showed the highest expression at P1 and the least reduction in expression levels at P4 and P10.

### 3.4. BACe56 Displays Increased Genome Stability and Retains GOI Expression

**Figure 6:**
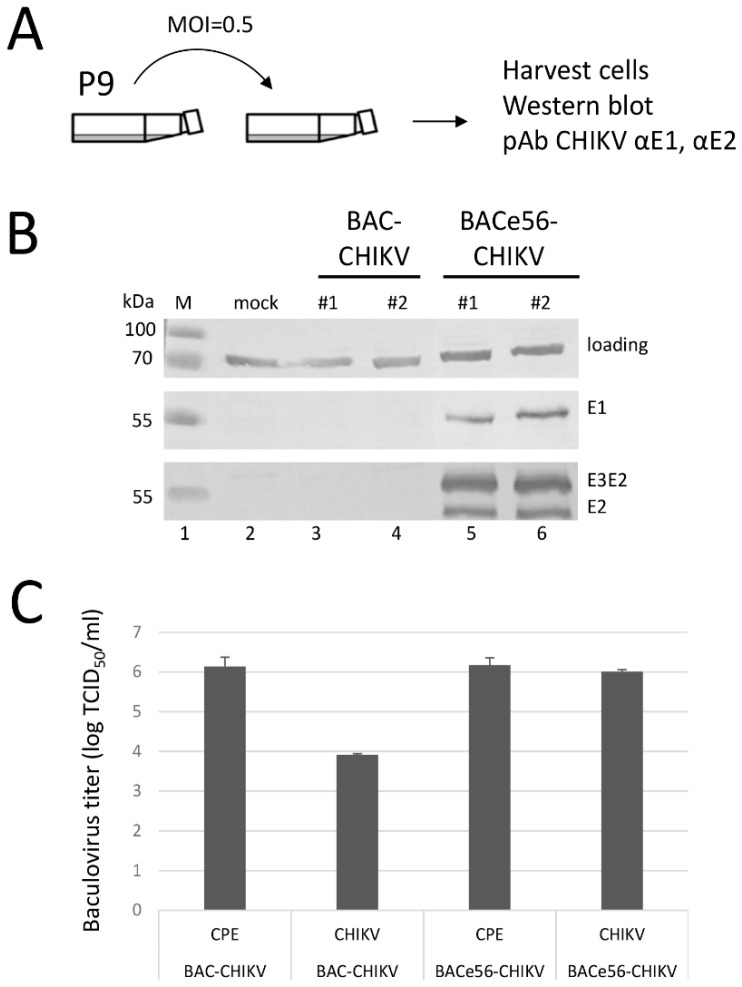
Stability of chikungunya VLP production with novel recombinant baculovirus BACe56. (**A**) Experimental set-up to determine VLP production after 10 duplicate passages. (**B**) Western blots detected with anti-E1 and anti E2 polyclonal antiserum show CHIKV glycoproteins E1, E2 and the precursor E3E2. #1 and #2 represent duplicate serial passage experiments. M: protein marker; loading: host cell protein; mock: healthy cells. (**C**) Titers of the recombinant baculovirus at passage 10. Viral titers were determined by end point dilution assay and are expressed as tissue culture infective doses 50% per ml (TCID_50_/_mL_). Titers were scored on cytopathic effect (CPE) or on reactivity with anti-E2 polyclonal antiserum (CHIKV).

To better compare the expression levels between the parental bacmid BAC-CHIKV and the novel bacmid BACe56-CHIKV, an infection experiment was conducted in duplicate and at an MOI of 0.5 TCID_50_ per cell, using P9 as inoculum (Figure 6A). In this way, a fair comparison of expression levels can be made. High expression levels of CHIKV envelope proteins (E3E2, E2 and E1) were detected for BACe56-CHIKV, but with the same substrate development time it was not possible to detect CHIKV envelope proteins with the parental bacmid BAC-CHIKV (Figure 6B). 

We hypothesized that the loss of CHIKV expression was the result of GOI deletion from BAC-CHIKV. In order to check whether the GOI had indeed been lost, the viral titers at P10 were determined in two ways: 1) by scoring the microtiter plate by cytopathic effects (CPE) and 2) by scoring after staining with anti-CHIKV E2 antibodies. If there is no loss of the GOI, the CPE and antibody-based titers are the same. If the GOI is lost, the antibody-based titers are lower than the CPE-based titers. The average titer of BAC-CHIKV was determined at 1.5 × 10^6^ (CPE) and 8.2 × 10^3^ (antibody) TCID_50_/_mL_, whereas BACe56-CHIKV titers were determined at 1.6 × 10^6^ (CPE) and 1.0 × 10^6^ (antibody) TCID_50_/_mL_ (Figure 6C). These results clearly demonstrate that, for BAC-CHIKV, the antibody-based titer is much lower than the CPE-based titer, meaning the relative proportion of baculoviruses with the GOI retained is less than 1%, whereas for BACe56-CHIKV the CPE- and antibody-based titers are similar. We conclude that BACe56-CHIKV is the most optimal bacmid generated, when both expression levels (Figure 4 and Figure 5) and stability of expression during serial passages (Figure 5 and Figure 6) are taken into account. 

### 3.5. BACe56 Expression Dynamics of a Chikungunya VLP Prototype Vaccine in Suspension Sf9 Cells

**Figure 7:**
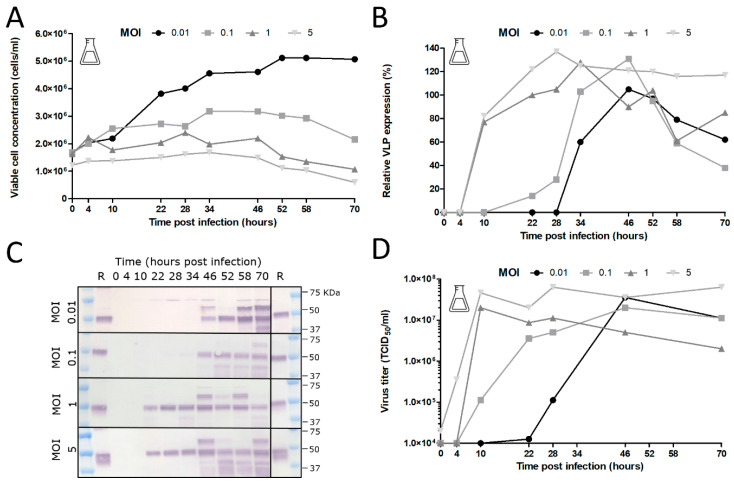
Effect of multiplicity of infection (MOI) on cell growth, baculovirus production and chikungunya VLP expression. (**A**) Cell growth after BACe56-CHIKV infection of Sf9 cells in shake flasks. Baculovirus infection was performed at different MOIs ranging from 0.01 to 5 TCID_50_/cell. (**B**) Baculovirus titers upon BACe56-CHIKV infection as function of MOI. Viral titers were determined by end point dilution assay and are expressed as tissue culture infective dose 50% per ml (TCID50/mL). (**C**) CHIKV VLP expression determined by Western blot detection with anti-E2 polyclonal antiserum. R; reference VLPs (**D**) Relative CHIKV VLP expression levels as function of MOI.

To further evaluate the potential of the improved BACe56 bacmid, its performance was investigated at different MOIs. Infections with BACe56-CHIKV were performed at MOIs of 0.01, 0.1, 1 and 5 TCID_50_/cell in shake flasks at a concentration 3 × 10^6^ Sf9 cells/mL (Figure 7). It was observed that the cultures infected with the lowest MOI reached higher maximum cell concentrations (of up to 5 × 10^6^ cells/mL), because uninfected cells were still able to divide (Figure 7A). Furthermore, infected cells had increased cell diameters and showed formation of CHIKV capsid bodies that appear in the nuclei as a result of CHIKV structural gene overexpression [28]. The baculovirus titers were also determined at several time points, which demonstrated that titer development was influenced by the initial MOI (Figure 7B). The infections at the higher MOIs of 1 and 5 TCID_50_/cell reached maximum baculovirus titers from 10 to 28 h post infection. The infections at MOI 0.01 and 0.1 TCID_50_/cell displayed a slight lag in the development of maximum baculovirus titers, which can be explained by the fact that the baculovirus needs additional round(s) of infection to infect all cells. The maximum titers are less dependent on the MOI and range from 10^7^–10^8^ TCID_50_/_mL_.

In order to determine heterologous protein expression levels, the CHIKV VLPs in the culture fluid were quantified by Western blot using anti-CHIKV-E2 polyclonal antiserum (Figure 7C). The infections at the higher MOIs (1 and 5 TCID_50_/cell) showed comparable expression of CHIKV VLPs over time with earlier and slightly higher maximum relative expression levels than with the lower MOIs (0.01 and 0.1 TCID_50_/cell) (Figure 7D). It was observed that the peak of CHIKV VLP accumulation was followed by a decrease, likely as a result of cell lysis and released proteases. This influenced the optimum time of harvesting, which was approximately 34 h post infection for the higher MOIs (of 1 and 5 TCID_50_/cell), and 46 h post infection for the lower MOIs (of 0.01 and 0.1 TCID_50_/cell). The CHIKV VLP concentrations at the optimum point of harvesting ranged between 2.6 and 3.8 mg/L.

**Figure 8:**
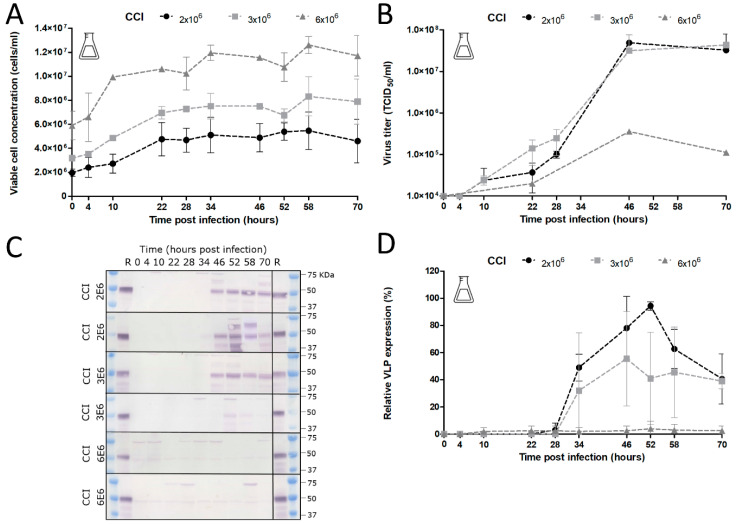
Effect of cell concentration at point of infection (CCI) on cell growth, baculovirus production and chikungunya VLP expression. (**A**) Cell growth after BACe56-CHIKV infection of Sf9 cells in shake flasks. Baculovirus infection was performed at MOI 0.01 TCID_50_/cell and different CCIs ranging from 2 × 10^6^ till 6 × 10^6^ cells/mL in shake flasks. Viral titers were determined by end point dilution assay and are expressed as tissue culture infective dose 50% per ml (TCID50/mL). Error bars are from duplicate experiments. (**B**) Baculovirus titers upon BACe56-CHIKV infection as function of CCI (**C**) CHIKV VLP expression determined by Western blot detection with anti-E2 polyclonal antiserum. R; reference VLPs. (**D**) Relative CHIKV VLP expression levels as function of CCI.

Next, the effect of cell concentration at time of infection (CCI) on CHIKV VLP expression was investigated (Figure 8). These experiments were performed with a low MOI since comparable product yields were reached for higher MOIs. The use of a low MOI is especially preferred for the development of a large-scale industrial production process as smaller quantities of virus are needed for the final production reactor. However, the use of low MOI requires multiple viral infection cycles to infect the entire cell population as not all cells become initially infected. Shake flask infection experiments with BACe56-CHIKV at aN MOI of 0.01 TCID_50_/cell were performed at a range of CCIs (2 × 10^6^, 3 × 10^6^ and 6 × 10^6^ cells/mL) to gain more insight into the interaction of the infection parameters for this particular production system. 

Similar patterns in cell growth were observed with specific growth rates and comparable cell-doubling factors for CCIs of 2 × 10^6^ and 3 × 10^6^ cells/mL (Figure 8A). The effect of CCI on baculovirus production and VLP expression was significant. Infections at a CCI of 2 × 10^6^ and 3 × 10^6^ cells/mL were quite similar, and both conditions demonstrated high baculovirus titers at 46 hpi (Figure 8B) and optimum VLP concentrations between 46 and 52 hpi (Figure 8C,D). In sharp contrast, the infection at a CCI of 6 × 10^6^ cells/mL displayed strongly reduced baculovirus titers (Figure 8B). No VLP expression was observed, neither in the medium fraction nor in the cell fraction (Figure 8C,D). We conclude that no successful baculovirus infection took place at a CCI of 6 × 10^6^ cells/mL and an MOI of 0.01 TCID_50_/cell.

### 3.6. BACe56 for Scale Up of CHIKV VLP Production in an Insect Cell Bioreactor

**Figure 9:**
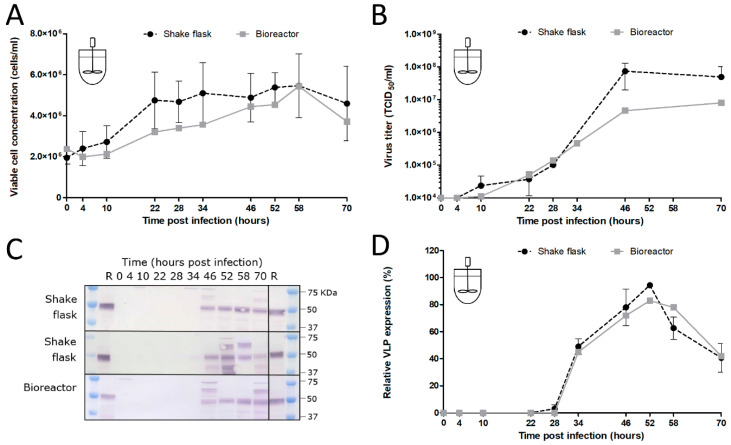
CHIKV VLP production in an insect cell bioreactor (**A**) Cell growth after BACe56-CHIKV infection of Sf9 cells in bioreactor compared to shake flask. Infections were performed at MOI 0.01 TCID_50_/cell and CCI 2 × 10^6^ cells/mL. (**B**) Baculovirus titers upon BACe56-CHIKV infection cells in bioreactor compared to shake flask. Viral titers were determined by end point dilution assay and are expressed as tissue culture infective dose 50% per ml (TCID50/mL). (**C**) CHIKV VLP expression determined by Western blot detection with anti-E2 polyclonal antiserum. R; reference VLPs (**D**) Relative CHIKV VLP expression levels in bioreactor compared to shake flask, determined by Western blot detection with anti-E2 polyclonal antiserum.

After the optimal MOI, CCI and VLP harvest time had been determined in shake flasks, the translation of these parameters to an Sf9 suspension cell bioreactor was investigated. First, uninfected Sf9 cells were cultured in the bioreactor. The controlled cultivation on larger scale did not have any influence on the cell growth as the uninfected cell culture showed the same growth characteristics and maximum cell density of roughly 1.1 × 10^7^ cells/mL as in shake flask cultivation. Next, an infection experiment was performed with BACe56-CHIKV at an MOI 0.01 TCID_50_/cell and a CCI of 2 × 10^6^ cells/mL. An initial lower growth rate was observed in the bioreactor as compared to the shake flasks, but the cell concentrations were similar from 46 hpi onwards (Figure 9A). The virus titer in the bioreactor developed at the same rate as in shake flasks until 28 hpi. After that, it somewhat slowed down and reached a lower maximum titer as compared to the shake flask of just under 10^7^ TCID_50_/_mL_ at 70 hpi (Figure 9B), which is probably a result of the reduced cell growth. Despite these minor differences in cell growth and baculovirus titers, the CHIKV VLP expression in the culture fluid was highly similar between bioreactor and shake flask experiments (Figure 9C,D). The optimum time of harvest in the bioreactor was estimated around 52 hpi. The CHIKV VLP concentration measured in the bioreactor was 2.1 mg/L. This experiment demonstrated that BACe56 can be successfully used for upscaling of the BEVS in an insect cell bioreactor.

## 4. Discussion

In this study, we scouted the AcMNPV genome for additional landing pads for the attTn7 transposition insertion site to present new options for the insertion of heterologous genes. These regions were initially selected based on data from a previous manuscript, which looked at direct insertion of recombinant protein coding regions into the baculovirus genome [21]. We chose several of the most interesting regions from that work to examine in the context of the Tn7 transposition system. Each region had a unique expression capacity and is, therefore, useful for future experimentation. Most notably, BACe56 (DE37) consistently produced higher levels of protein expression than the control bacmid strain, with some proteins generating 1.4 times the original levels of recombinant protein. This suggests that some aspects of this location, whether due to the stability of the insertion or to some unknown transcriptional enhancer, lead to higher levels of protein production. While a 1.4 time increase in yield might appear to be minimal at a first glance, such an improvement in the production of proteins for therapeutic usage or as a vaccine product could carry significant reductions in cost of operations or the scale of production required. 

Two of the other strains, DE38 (BAC51/52) and DE40 (v-Ubi/39k), with similar expression levels to the original polh locus, could potentially be used as alternative sites for the introduction of heterologous genes, or might prove useful as secondary insertion locations if the expression of multiple genes is desired. While the 2x reduced expression from the DE39 (gp37/DNApol) strain may seem to be a negative attribute of this location, in the case of slightly toxic proteins, or proteins which suffer from aggregation or solubility issues when overexpressed, the reduced expression level of DE39 might prove a good solution. 

The serial undiluted passaging experiments very clearly showed that it was beneficial to separate the GOI from the unstable mini-F replicon in the bacmid. Based on the results, we could conclude that GOI expression from the odv-e56 (pif-5) locus resulted in the highest overall expression levels and increased stability upon serial passage compared to the original bacmid. The loss of GOI expression upon serial passage of original bacmid constructs was more rapid than what was seen in an earlier study [19]. However, the size of the GOI in this study (CHIKV structural genes, 3.8 kbp) is much larger than the GFP gene (0.7 kbp), which increases the chances of deletion. Given the non-essential nature of the miniF replicon for baculovirus infection, the enhanced GOI stability in BACe56 vs. the original bacmid most likely relates to the relocation of the attTn7 site to a more stable location in the genome. In fact, odv-e56 is located in between baculovirus essential genes (ie-1 and ie-2) that cannot be deleted. Furthermore, instability of certain regions may correlate with a low density of baculovirus homologous repeat regions (hrs), which are dispersed throughout the baculovirus genome and are believed to act as origins of viral DNA replication (oris) [29]. The odv-e56 gene is located quite close to hr1, which may enhance its stability in the genome and perhaps influences the transcriptional activity of nearby genes by functioning as an enhancer. This may explain why BACe56 gives slightly higher recombinant protein yields.

As the demand for the production of recombinant protein complexes increases, the four new bacmids generated in this study will be useful tools for optimizing expression and quality, while remaining the advantages of expression during the very late stage of the lytic cycle. Although these next-generation bacmids have been developed to be compatible with the Bac-to-Bac system, they can be used in combination with any Tn7-based baculovirus cloning method. 

The added stability of GOI expression upon serial undiluted passage of BACe56 could have major impacts on its utility for large-scale protein production, which we examined in shake flask and bioreactor experiments. The interplay between MOI, CCI and time of harvest (TOH) of the new bacmid BACe56-expressing CHIKV VLPs in Sf9 suspension cells was investigated. Overall, the reported results showed a clear difference between infections at high MOIs (1 and 5) and low MOIs (0.01 and 0.1), which is in line with other studies [30,31,32,33]. Infection of the insect cells at lower MOIs lead to 1–2 infection cycles in the culture and to the proliferation of the uninfected cells and thus higher maximum cell concentrations. Infection at an MOI of five immediately inhibited cell growth because all cells were infected simultaneously. The differences in cell growth, viral infection process and VLP expression observed between infections at diverse MOIs highlights the importance of this infection parameter in the establishment of a production process for recombinant baculovirus-expressed VLPs. Infection at an MOI of 0.01 is preferred for further process developments, since comparable product yields were reached because higher MOIs and smaller quantities of virus are required, which may obliviate the use of additional bioreactors for virus production.

The choice of CCI is also very important since it can cause significant alterations in product yield, especially when low-MOI infection is applied. Baculovirus infection above a certain optimum CCI leads to an ineffective infection with poor baculovirus titers and low product concentrations [32,34,35]. Indeed, we observed unsuccessful infection at a CCI above 4 × 10^6^ cells/mL with no detectable CHIKV VLP yields. The optimum VLP production was achieved at a CCI of 2 × 10^6^ cells/mL, whereas infections at CCIs higher than 3 × 10^6^ cells/mL resulted in a reduction in baculovirus infection and VLP production. With regard to specific protein yields, we generated overall CHIKV VLP yields between 2–4 mg/L. In similar systems, the yields varied from 0.2 mg/L for SARS coronavirus VLPs [36] to 662 mg/L for rotavirus VLPs [37]. Most likely, further optimization of CHIKV VLP yields can be accomplished since CHIKV VLPs up to 40 mg/L were produced in adherent Sf21 cells [22], while 28 mg/L CHIKV VLPs were produced in in Sf21-derived suspension Sf-basic cells [38]. We conclude that BACe56 is a stable vector with improved expression characteristics that is suitable for the production of complex VLP vaccines in insect cell bioreactors.

## Figures and Tables

**Table 1:** attTn7 location in different strains.

Strain	Location	Left Flanking Sequence	Right Flanking Sequence
DE32	polh locus	TGCTTCCGGCTCGTATGTTG	TCCCCCTTTCGCCAGCTGGC
DE37	mid odv-e56	TTGACGACAAGTGCGCTGCA	ATAACAAGCAGGCCTCGGCG
DE38	between orf51 and orf52	GTTTTTTTCTAGTGTCGTACTT	TTTTACAATGCGTCTGTTGTCC
DE39	between v-ubiquitin and 39k	AATAATAAAAACCATTAAAT	ACAAAAGTTTTTTATT
DE40	between gp37 and DNApol	TTGGTCAAAAACGTTATGTT	GAAACATAATAACACCTTAC
